# Antenatal Maternal Long-Term Hypoxia: Acclimatization Responses with Altered Gene Expression in Ovine Fetal Carotid Arteries

**DOI:** 10.1371/journal.pone.0082200

**Published:** 2013-12-18

**Authors:** Ravi Goyal, Jonathan Van Wickle, Dipali Goyal, Nathanael Matei, Lawrence D. Longo

**Affiliations:** 1 Center for Perinatal Biology, School of Medicine, Loma Linda University, Loma Linda, California, United States of America; 2 Department of Basic Sciences, School of Medicine, Loma Linda University, Loma Linda, California, United States of America; Université de Montréal, Canada

## Abstract

In humans and other species, long-term hypoxia (LTH) during pregnancy can lead to intrauterine growth restriction with reduced body/brain weight, dysregulation of cerebral blood flow (CBF), and other problems. To identify the signal transduction pathways and critical molecules, which may be involved in acclimatization to high altitude LTH, we conducted microarray with advanced bioinformatic analysis on carotid arteries (CA) from the normoxic near-term ovine fetus at sea-level and those acclimatized to high altitude for 110+ days during gestation. In response to LTH acclimatization, in fetal CA we identified mRNA from 38 genes upregulated >2 fold (P<0.05) and 9 genes downregulated >2-fold (P<0.05). The major genes with upregulated mRNA were SLC1A3, Insulin-like growth factor (IGF) binding protein 3, IGF type 2 receptor, transforming growth factor (TGF) Beta-3, and genes involved in the AKT and BCL2 signal transduction networks. Most genes with upregulated mRNA have a common motif for Pbx/Knotted homeobox in the promoter region, and Sox family binding sites in the 3′ un translated region (UTR). Genes with downregulated mRNA included those involved in the P53 pathway and 5-lipoxygenase activating proteins. The promoter region of all genes with downregulated mRNA, had a common 49 bp region with a binding site for DOT6 and TOD6, components of the RPD3 histone deacetylase complex RPD3C(L). We also identified miRNA complementary to a number of the altered genes. Thus, the present study identified molecules in the ovine fetus, which may play a role in the acclimatization response to high-altitude associated LTH.

## Introduction

The cellular and molecular mechanisms by which an organism acclimatizes to high altitude long-term hypoxia (LTH) are complex and not well understood. In human pregnancy, antenatal LTH, as a consequence of high altitude residence, maternal smoking, anemia, heart and/or lung diseases, placental pathologies (such as pre-eclampsia, placental insufficiency, abruptio placenta), or other factors may be associated with a generalized intrauterine growth restriction (IUGR) reduced organ weights (including brain and heart), and/or persistent pulmonary hypertension of the newborn [1]. In rodents, antenatal hypoxia also is known to cause reduced litter size and low birth weight pups [2].

In previous studies we have demonstrated that in response to prolonged maternal antenatal hypoxic exposure at high altitude (3,801 m). The sheep fetus undergoes successful acclimatization without the LTH-induced pathological conditions observed in many other species [3], [4]. This also has been reported in the sheep by others [5]. In contrast, high-altitude (2,740 to 3,100 m) exposed human infants have been reported to demonstrate intrauterine growth restriction, reduced weight of brain and other organs, increased incidence of pulmonary hypertension, as well as other pathological conditions [6], [7]. Of note, studies suggest 100 g of birth-weight reduction per 1000 m of elevation [8]. Despite the higher elevation in our study (3,801 m), the influence of hypoxic stress on the sheep fetus is attenuated, representing a unique adaptive characteristic.

Furthermore, the fetuses acclimatized to high altitude LTH had near-normal [9] cerebral blood flow despite a significant 27±4% decrease in cardiac output, and a 49±6% decrease in blood flow to the carcass and most other organs [3]. Additionally, we have observed that the acclimatized ovine fetus can maintain normal brain weight, cerebral oxygenation, cerebral O_2_ metabolic rate, sagittal sinus PO_2_, cortical tissue PO_2_, and electro-encephalographic activity at a level similar to the sea-level normoxic fetus [9], [10]. These findings raise questions regarding the mechanisms whereby, in contrast to the human, the ovine fetus successfully maintains normal CBF and brain weight during prolonged hypoxic exposure.

Carotid arteries (CA) have been shown to play a crucial role in the regulation and maintenance of CBF [11]. During increased flow demand, there is a significant pressure gradient from CA to cerebral arteries [12].. Importantly, other studies suggest that much of the change in systemic pressure results in dilation/contraction of the large arteries that supply the brain [13]. These studies underscore the importance of CA in the regulation of CBF, and suggest that failure of CA to effectively regulate the pressure of the blood reaching delicate cerebral arteries may lead to rupture with hemorrhage. Moreover, evidence suggests that the large cranial arteries of premature as well as intrauterine growth restricted infants may be unable to regulate effectively their CBF, as opposed to the near-term newborn [14], [15].

Thus, we tested the hypothesis that in fetal sheep, antenatal maternal high altitude long-term hypoxia is associated with changes in the gene expression in carotid arteries, which may be important in successful cerebrovascular acclimatization response and the maintenance of CBF, as well as normal for brain growth. By use of novel ovine oligonucleotide microarrays and signal pathway analysis, in near-term (140 days gestation) fetuses from both normal sea-level controls and those acclimatized to high altitude during antenatal development. We examined changes in carotid arteries gene expression pathways. We also conducted advanced bioinformatic analysis to identify *cis*-regulatory elements, trans-acting factors, and microRNA (miRNA), which can regulate gene expression in the CA.

## Methods

### Experimental animals and tissues

All experimental procedures were performed within the regulations of the Animal Welfare Act, the National Institutes of Health Guide for the Care and Use of Laboratory Animals, and - the present study was approved by the Loma Linda University Animal Care and Use Committee.

Eight “Western breed” ewes were obtained from a single supplier (Nebeker Ranch, Lancaster, CA) and were allocated to two groups of four each, control or long-term hypoxic groups. For control (normoxic) group, sheep were maintained at the suppliers ranch (a 300 m elevation) on alfalfa pellets ad libitum. For the hypoxia study, at 30 days gestation, ewes were transported to the Barcroft Laboratory, White Mountain Research Station (WMRS, CA; elevation 3,801 m, barometric pressure −480 Torr), where they were kept until 135 days gestation. They were maintained in a sheltered outdoor pen and were fed with alfalfa pellets ad libitum. Sheep in both groups were kept in natural day-night conditions. In previous studies, we have obtained mean maternal arterial blood gas values from 12 adult sheep at WMRS. These were pO2 = 60±5 Torr, PCO2 = 30.0±2.5 Torr, and pH = 7.36±0.06. In contrast normoxic control sheep (elevation 346 m) had pO2 of 100±5 Torr, pCO2 35.2±0.9, pH = 7.44±0.1. Importantly, with LTH exposure, fetal arterial PO_2_ fell from the normoxic value of 29.7±2.1 to 19.1±2.1 Torr. At 135 days gestation, ewes from both groups were transported (4 to 7 hrs trip) to our laboratory at Loma Linda University. Soon after arrival, surgeries were conducted to isolate fetal carotid arteries. In the hypoxic group, shortly following arrival, a tracheal catheter was placed in the ewe, through which N_2_ flowed at a rate adjusted to maintain its PaO_2_ at ∼60 Torr, similar to the levels achieved at high altitude. For both groups, pregnant ewes were anesthetized with thiopental sodium (10 mg.kg^−1^, i.v.), and anesthesia was maintained with inhalation of 1% isoflurane in oxygen throughout surgery. The fetus was delivered by hysterotomy and following the removal of carotid arteries, the fetus and ewe was euthanized with an overdose of the proprietary euthanasia solution, Euthasol (pentobarbital sodium 100 mg.Kg^−1^ and phenytoin sodium 10 mg.Kg^−1^; Virbac, Ft. Worth, TX). Studies were performed in isolated mid-carotid artery segments cleaned of endothelium, adipose, and connective tissue.

### Tissue Collection and Microarray Processing

We have described this technique in detail [16]. Ovine oligonucleotide microarrays were obtained from Agilent Technologies (Santa Clara, CA) and analysis was conducted by utilizing the commercial services of GenUs Biosystems (Northbrook, IL). Briefly, tissue samples were lysed in Tri-reagent (Ambion, Austin, TX) and total RNA was isolated using phenol/chloroform extraction, followed by purification over spin columns (Ambion). The concentration and purity of total RNA were measured by spectrophotometry at OD260/280, and the quality of the total RNA sample was assessed using an Agilent Bioanalyzer with the RNA6000 Nano Lab Chip (Agilent Technologies).

Labeled cRNA was prepared by linear amplification of the Poly(A)+ RNA population within the total RNA sample. Briefly, 1 µg of total RNA was reverse transcribed after priming with a DNA oligonucleotide containing the T7 RNA polymerase promoter 5′ to a d(T)24 sequence. After second-strand cDNA synthesis and purification of double-stranded cDNA, *in vitro* transcription was performed using T7 RNA polymerase. The quantity and quality of the labeled cRNA were assayed by spectrophotometry and the Agilent Bioanalyzer.

One µg of purified cRNA was fragmented to uniform size and applied to Agilent Sheep Gene Expression Microarray, 8×15K (Design ID 019921, Agilent Technologies) in hybridization buffer. Arrays were hybridized at 65°C for 17 hrs. in a shaking incubator and washed at 37°C for 1 min. Rinsed and dried arrays were scanned with an Agilent G2565 Microarray Scanner (Agilent Technologies) at 5 µm resolution. Agilent Feature Extraction software was used to process the scanned images from arrays (grid and feature intensity extraction) and the data generated for each probe on the array was analyzed with Gene Spring GX v7.3.1 software (Agilent Technologies). Annotations are based on the Agilent eArray annotation file dated January 2010. The data set has been submitted in GEO database, the accession no. Is GSE49920.

### Pathway/Network Analysis

Each gene was annotated/checked manually using NCBI Blast Search, Unigene, Entrez or other databases. Genes with unknown sequences for *Ovis aries* were annotated if more than 90% sequence homology was identified with *Bos taurus*. We then analyzed the annotated genes using an Ingenuity Pathway Analysis Program (Ingenuity Systems, Redwood City, CA). Group of genes were analyzed by Meme Software Suite to examine common motifs and *cis*-regulatory elements [17]. Common motifs and *cis*-regulatory elements were analyzed by Transfac Public 6.0 to examine transcription factors [18]. The 3′ un-translated region (3′UTR) of the group of upregulated and downregulated regions were examined by Diana Lab Softwares (Athens, Greece) [19].

### Real Time PCR Validation

To validate the results of the microarray analysis, we examined expression of IGFBP3, AKT1, CRABP2, ERK1, and ERK2 genes by using real time PCR. Using the same probe sequences as those on the microarray chip, we designed primers with the use of Primer 3 web-based software (Primer3 website. Available: http://frodo.wi.mit.edu/primer3/. Accessed 2013 November 1). The primers were synthesized by Integrated DNA technologies (Coralville, CA). The total RNA (1 ug per reaction) was reverse transcribed using Quantitect reverse transcriptase kit (Qiagen, Valencia, CA). Relative expression was normalized to 18S RNA and fold-changes were calculated using the ΔΔCt method with normalization of individual PCR efficiencies [20]. Samples (n = 4 from each group) were analyzed on the Roche LightCycler 1.5 (Roche, Indianapolis, IN).

### Western Immunoblot Validation

Isolated carotid arteries from normoxic and hypoxic fetuses (n = 4 in each group) were cleaned of adventitia and the endothelium was denuded. These arteries were homogenized with a tissue grinder in ice-cold cell lysis buffer (Cell Signaling Technology, Danvers, MA), as we have described [21]. Protein concentrations were measured using a protein assay kit (Bio-Rad Laboratories, Hercules, CA) with bovine serum albumin (BSA) as a reference protein. A Mini Trans-Blot Electrophoretic Transfer Cell system (Bio-Rad Laboratories) was used to transfer proteins from the gel to a nitrocellulose membrane at 100 V for 3 h. We then performed an overnight incubation of AKT and IGFBP3 specific primary antibodies (1∶500 dilution). We used the total ERK as an internal control for equal protein loading, as well as the blocking peptide for each subtype specific antibody as a negative control. All antibodies were obtained from Abcam Inc. (Cambridge, MA). Earlier, we have demonstrated that total ERK levels do not change in carotid arteries in response to long-term high altitude acclimatization [22]. The membrane then was incubated in chemiluminescence luminol reagent (Pierce, Rockford, IL) for 1 min, and the protein band detected using an Alpha Innotech Chemiluminescent imaging system (San Leandro, CA).

### Epiflorescence Imaging Studies Validation

By use of an Evos Florescence Microscope (Advanced Microscopy Group, Bothell, WA) using standard techniques, we examined the expression of PKNOX1. Alpha smooth muscle actin and nuclear stain Dapi (4′, 6-Diamindino-2-Phenylindole -di-Lactate) were used as controls as we have reported [23]. Briefly, arterial segments (n = 4 from each group) were sliced to 10 μ sections using a Leica Cryostat (Leica Microsystems Inc. Buffalo Grove, IL). Tissue sections were then sealed on microscopic slides with Vectashield mounting medium containing DAPI (H-1200, Vector Labs, Burlingame, CA). All antibodies were obtained from Abcam Inc. Images were analyzed using the ImageJ software (NIH), and protein expression was measured as fluorescent intensity/unit area normalized to the florescent intensity/unit area of the alpha smooth muscle actin control.

### Statistics

To compare individual expression values across arrays, the raw intensity data from each gene was normalized to the 75^th^ percentile intensity of each array. Only genes with values greater than background intensity for all samples within each group were used for further analysis. For the microarray data analysis, we used a parametric T-test that assumes variances are **not** equal (Welch T-test). This test also assumes that the sheep were selected from a **population** that follows a normal distribution of individual gene expression. To further ensure individual gene data fit a normal distribution, all T-test p-values were calculated using the means of log10 transformed values. Differentially expressed genes were identified by 2-fold change and Welch T-test [24] p-values<0.05 between each treatment group and its age-specific normoxic control. Statistical significance in the real-time PCR, western immunoblot and immuno histochemistry was determined by Students t-test (P<0.05).

## Results

As a consequence of antenatal maternal long-term hypoxia, we did not observe a significant reduction in the fetal body or brain weight at 140 days of gestation (data not shown), and this was similar to our previous studies [4]. In terms of gene regulation, antenatal maternal high altitude long-term hypoxic exposure was associated with 38 genes being upregulated more than 2-fold (P<0.05; Table 1) and 9 genes downregulated more than 2-fold (P<0.05; Table 2).

**Table 1:** Top upregulated genes in high altitude acclimatized ovine fetal carotid arteries compared to normal control.

Symbol	Entrez Gene Name	Fold Change	FH Mean	FH SD	FN Mean	FN SD	p-value
SLC16A3	Solute carrier family 16, member 3 (monocarboxylic acid transporter 4)	6.866	0.38	0.28	0.06	0.03	1.77E-02
TNC	Tenascin C	5.704	4.72	3.49	0.83	0.38	2.47E-02
IGFBP3	Insulin-like growth factor binding protein 3	5.288	1.20	0.12	0.23	0.13	3.04E-02
TP53I11	Tumor protein p53 inducible protein 11	5.133	0.13	0.08	0.03	0.02	4.46E-02
IGF2R	Insulin-like growth factor 2 receptor	4.906	0.59	0.41	0.12	0.04	2.99E-02
ATF3	Activating transcription factor 3	4.384	0.43	0.14	0.10	0.05	1.82E-02
SERPINH1	Serpin peptidase inhibitor, clade H (HSP47; collagen binding protein 1)	4.246	12.89	5.76	3.03	0.92	1.13E-02
PIM2	Pim-2 oncogene	3.489	0.07	0.01	0.02	0.01	3.40E-03
DCAF8	DDB1 and CUL4 associated factor 8	3.374	0.45	0.22	0.13	0.06	3.13E-02
TGFB3	Transforming growth factor, beta 3	2.919	1.43	0.50	0.49	0.26	4.56E-02
MYOT	Myotilin	2.789	5.77	1.12	2.07	0.46	3.98E-03
DNAJC21	DnaJ (Hsp40) homolog, subfamily C, member 21	2.613	0.23	0.05	0.09	0.03	1.38E-02
CRELD2	Cysteine-rich with EGF-like domains 2	2.597	0.43	0.16	0.17	0.02	4.07E-02
BCL2	B-cell CLL/lymphoma 2	2.549	0.85	0.24	0.33	0.16	4.83E-02
GNL1	Guanine nucleotide binding protein-like 1	2.52	0.35	0.10	0.14	0.06	4.20E-02
HR	Hairless homolog (mouse)	2.499	0.17	0.02	0.07	0.02	9.53E-03
SNRNP70	Small nuclear ribonucleoprotein 70 kDa (U1)	2.485	1.12	0.46	0.45	0.10	3.78E-02
ATF6B	Activating transcription factor 6 beta	2.386	0.29	0.10	0.12	0.04	3.48E-02
SURF6	Surfeit 6	2.351	0.21	0.06	0.09	0.04	4.88E-02
KDM4A	Lysine (K)-specific demethylase 4A	2.327	0.42	0.11	0.18	0.04	1.17E-02
ACTN1	Actinin, alpha 1	2.326	23.45	2.57	10.08	1.35	1.24E-03
DYNC1H1	Dynein, cytoplasmic 1, heavy chain 1	2.307	3.16	0.49	1.37	0.53	4.59E-02
MED16	Mediator complex subunit 16	2.264	0.14	0.05	0.06	0.02	4.97E-02
ADIPOR1	Adiponectin receptor 1	2.247	0.39	0.08	0.17	0.05	2.31E-02
DNAJB5	DnaJ (Hsp40) homolog, subfamily B, member 5	2.247	1.24	0.47	0.55	0.12	4.20E-02
MTMR2	Myotubularin related protein 2	2.231	0.22	0.08	0.10	0.03	4.78E-02
MTCH1	Mitochondrial carrier 1	2.224	5.12	1.75	2.30	0.35	3.63E-02
CDV3	CDV3 homolog (mouse)	2.206	0.46	0.15	0.21	0.07	4.51E-02
KHSRP	KH-type splicing regulatory protein	2.153	0.27	0.07	0.13	0.02	1.34E-02
RBM42	RNA binding motif protein 42	2.153	1.91	0.50	0.89	0.30	3.58E-02
ATG4B	ATG4 autophagy related 4 homolog B (S. cerevisiae)	2.119	1.46	0.46	0.69	0.10	3.55E-02
WASF2	WAS protein family, member 2	2.054	0.40	0.09	0.19	0.05	1.88E-02
COL18A1	Collagen, type XVIII, alpha 1	2.05	6.00	0.60	2.93	0.54	1.45E-02
PLXNB1	Plexin B1	2.047	0.56	0.10	0.27	0.09	4.24E-02
COX7B	Cytochrome c oxidase subunit VIIb	2.045	0.64	0.15	0.31	0.09	3.01E-02
ANXA11	Annexin A11	2.026	0.93	0.21	0.46	0.16	4.46E-02
FADS2	Fatty acid desaturase 2	2.023	0.59	0.20	0.29	0.06	4.68E-02
AKT1	v-akt murine thymoma viral oncogene homolog 1	2.019	5.00	0.52	2.48	0.52	1.41E-02

**Table 2:** Top downregulated genes in high altitude acclimatized fetal carotid arteries compared to normal control.

Symbol	Entrez Gene Name	Fold Change	FH Mean	FH SD	FN Mean	FN SD	p-value
BRB/BRN-2	Brain Ribonucleas	−3.63	4.10	0.73	14.91	6.33	0.01
ALOX5AP	Arachidonate 5-lipoxygenase-activating protein	−3.247	0.11	0.03	0.37	0.04	0.007
RNASE6	Ribonuclease, RNase A family, k6	−2.924	0.38	0.04	1.12	0.44	0.039
TRA@	T cell receptor alpha locus	−2.625	0.11	0.04	0.30	0.11	0.023
CRABP2	Cellular retinoic acid binding protein 2	−2.584	0.16	0.07	0.42	0.08	0.039
RPL35	Ribosomal protein L35	−2.257	1.87	0.19	4.21	0.42	0.003
RGS10	Regulator of G-protein signaling 10	−2.123	0.16	0.06	0.34	0.07	0.043
BATF3	Basic leucine zipper transcription factor, ATF-like 3	−2.096	0.10	0.03	0.20	0.06	0.038
FAM35A	Family with sequence similarity 35, member A	−2.008	0.30	0.06	0.59	0.11	0.013

As shown in Table 3, based on Ingenuity Pathway Analysis, the major functional networks altered were those associated with cell survival, cellular development, cellular growth and proliferation, cell morphology, cardiovascular system development and function, and hematological system development and function. Also, shown are the five major canonical pathways significantly altered because of antenatal maternal LTH acclimatization.

**Table 3:** Chief Functional and Canonical Pathways Altered by Long-Term Hypoxia.

Functional Pathways	Molecules
Cell Survival	ATF3, ATG4B, IGF2R, ADIPOR1, BCL2, DYNC1H1, AKT1, CRABP2, RGS10, IGFBP3, TGFB3, PLXNB1, COL18A1, TRA@, PIM2
Cellular Development	ATF3, TNC, IGF2R, BCL2, DYNC1H1, AKT1, BATF3, RGS10, TGFB3, IGFBP3, PLXNB1, WASF2, COL18A1, TRA@
Cellular Growth and Proliferation	ATF3, TNC, SURF6, TP53I11, MTCH1, HR, ADIPOR1, IGF2R, BCL2, DYNC1H1, AKT1, ANXA11, SERPINH1, CRABP2, TGFB3, FADS2, IGFBP3, PLXNB1, WASF2, COL18A1, ACTN1, TRA@, PIM2
Cell Morphology	ATF3, TNC, AKT1, SERPINH1, ATG4B, TGFB3, IGFBP3, WASF2, COL18A1, TRA@, PIM2, BCL2
Cardiovascular System Development and Function	TNC, ATF3, AKT1, SERPINH1, TGFB3, IGFBP3, PLXNB1, WASF2, COL18A1, IGF2R, PIM2, BCL2
Hematological System Development and Function	TNC, ATF3, AKT1, BATF3, RGS10, PLXNB1, WASF2, IGF2R, TRA@, PIM2, BCL2
**Canonical Pathways**	**Molecules**
VEGF Signaling	AKT1,ACTN1,BCL2
PTEN Signaling	AKT1, IGF2R, BCL2
PI3K Signaling in B Lymphocytes	AKT1, ATF3, ATF6B
TGF-β Signaling	TGFB3, BCL2
PI3K/AKT Signaling	AKT1, BCL2

**Figure 1:**
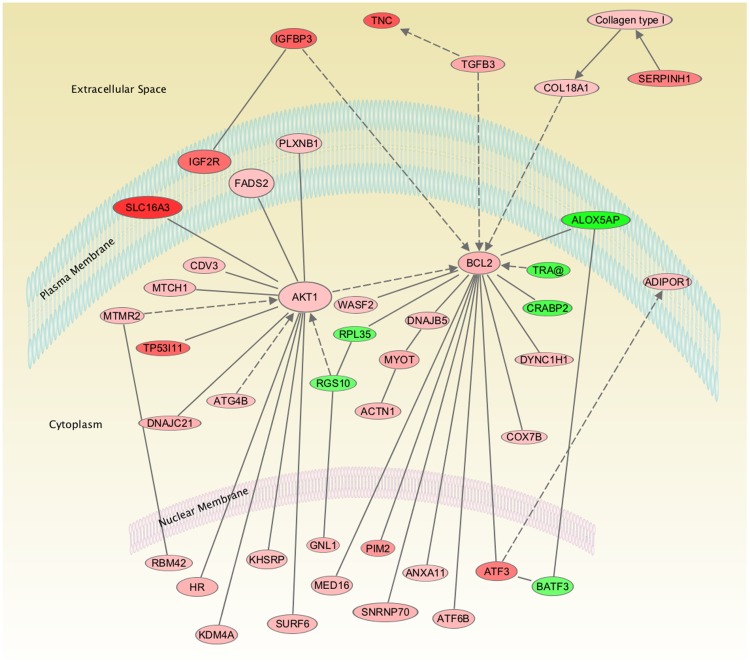
Demonstrates the network of genes in ovine fetal carotid arteries extra-cellular, plasma membrane, cytoplasm, and nucleus altered as a consequence of antenatal maternal long-term hypoxia. Red color represents the upregulated genes; the higher intensity of color denotes greater fold-change; green represents the downregulated genes.

**Figure 2:**
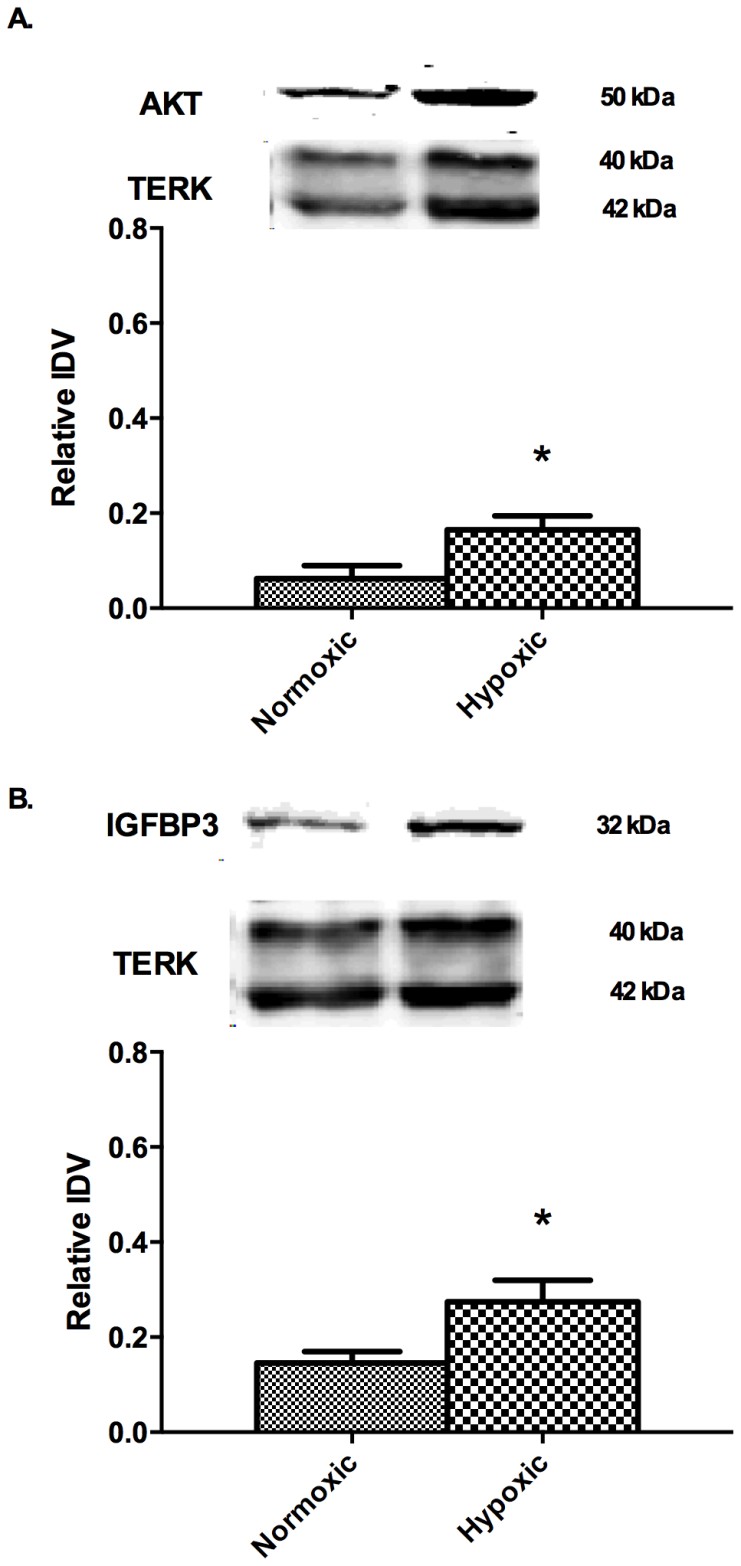
Demonstrates protein expression of (A) AKT and (B) IGFBP3. N = 4 in each group; Total extracellular regulated kinase (TERK) served as an internal control to which AKT and IGFBP3 were normalized to provide relative integrated density (IDV). * Denotes significant changes in protein expression by Students t-test (P<0.05); FN - Carotid arteries from control (normoxic) fetus, FH - Carotid arteries from antenatal long-term hypoxia acclimatized animals.

**Figure 3:**
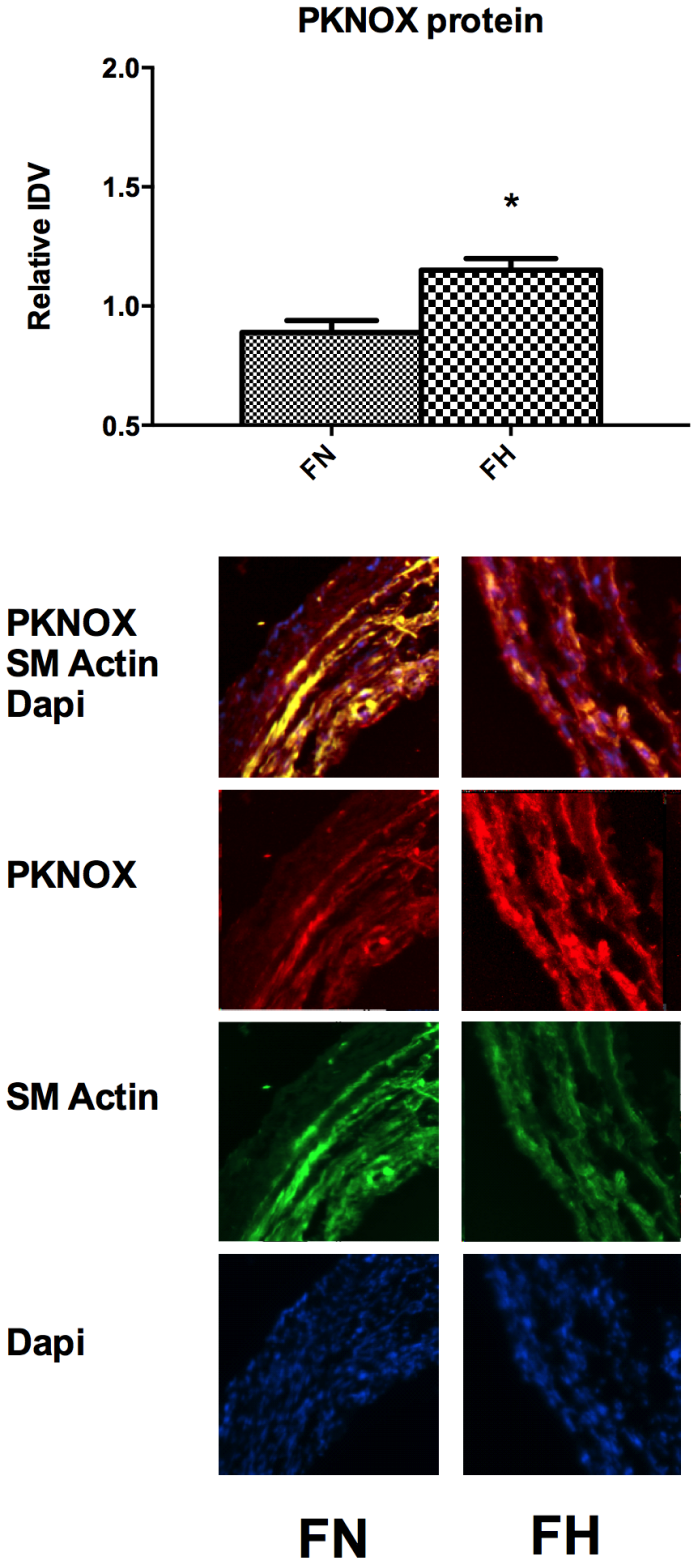
Demonstrates immunohistochemistry images of PKNOX1 expression in carotid arteries from normoxic fetus (FN) and hypoxic fetuses (FH). *Denotes statistically significant difference by Students t-test (P<0.05).

As shown in Figure 1, analysis of the genes involved in the altered functional and canonical pathways belong to two major networks of genes namely AKT1 and B-cell lymphoma 2 (BCL2) pathways. To confirm the findings of microarray data, few genes with moderate changes in fold-change were examined with real-time PCR analysis. Real-time PCR analysis, demonstrated that Insulin like growth factor binding protein 3 (IGFBP3) was upregulated 3.6±1.2 and AKT was upregulated 2.1±0.3 - fold and were similar to the findings in the microarray analysis. ERK1 and 2 did not show any significant changes with LTH with real-time PCR or microarray analysis, this was further confirmed with western immunoblot analysis and used a loading control. Also, with real-time PCR, CRABP2 showed 2.3±0.4 -fold downregulation on long-term hypoxic exposure. Similarly, total ERK (TERK) and α-smooth muscle actin showed no significant change on western immunoblot (Figure 2) and immuno histochemistry (Figure 3) analysis. Further validation of AKT (Figure 2A) and IGFBP3 (Figure 2B) demonstrated an increased level of expression by western immunoblot. Additionally, PKNOX analysis by immuno histochemistry (Figure 3) analysis demonstrated a significantly increased expression.

### Analysis of 1800 bp upstream of transcription start site (TSS) of up- and downregulated genes

**Figure 4:**
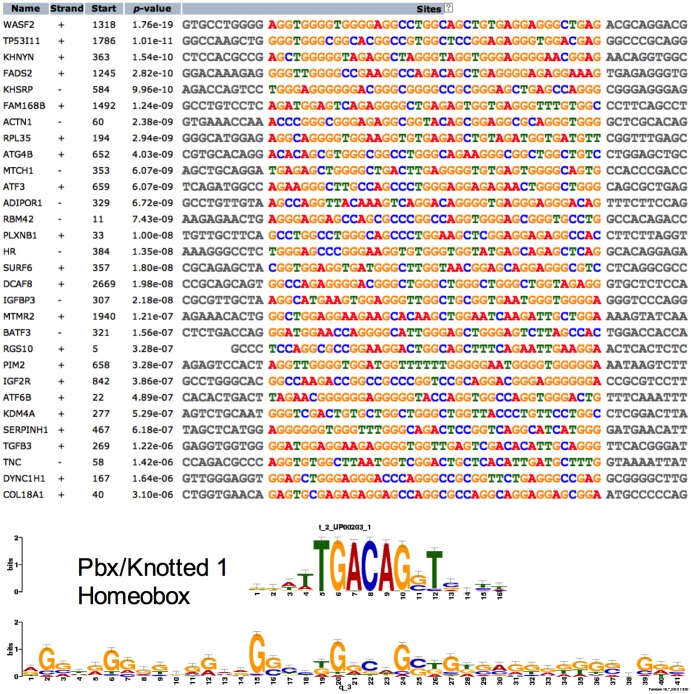
Demonstrates a common motif in 1800 base pairs upstream to the transcription start site of the upregulated genes. Strand + and − denotes the sense and antisense DNA sequence. The start denotes the number of base pair upstream to the transcriptional start site. p-value denotes the probability of the presence of motif by chance in the given sequence as a part of group of sequence altered by long-term hypoxic exposure. The bottom of the figure shows a cis-regulatory element for Pbx/knotted 1 homeobox transcription factor, which can bind on the common motif of the genes shown above. Bits refer to the likely hood of the presence of a particular letter of a specific motif, at a specific site, in more number of background sequences of genes altered by long-term hypoxia.

**Figure 5:**
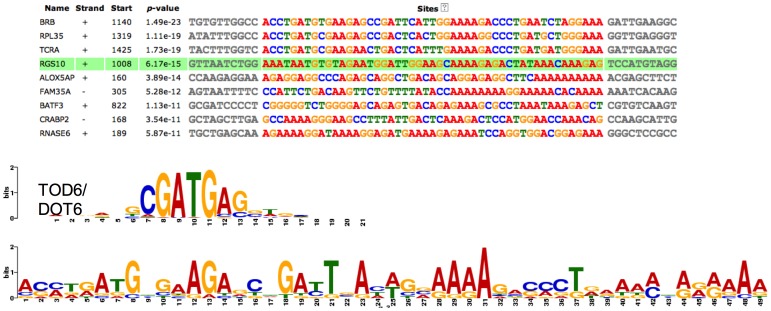
Demonstrates a common motif in 1800 base pairs upstream to the transcription start site of the downregulated genes. The bottom of the figure shows a cis-regulatory element for TOD6/DOT6 transcription factor, which can bind on the common motif of the genes shown above.

As shown in Figure 4, we found a 41 bp common motif in the upregulated gene sequence. Notably, TGFB and Pbx/Knotted 1 Homeobox binding sites are present in the promoters of a number of upregulated genes. The promoter regions of the 9 downregulated genes demonstrate a common motif sequence of 49 bp (Figure 5). In this 49 bp region, we identified binding sites for transcription factors DOT6 and TOD6, components of the RPD3 histone deacetylase complex RPD3C(L) responsible for the deacetylation of lysine residues on the N-terminal part of the core histones (H2A, H2B, H3 and H4).

### Analysis of the 3′ un-translated region of the altered genes

**Figure 6:**
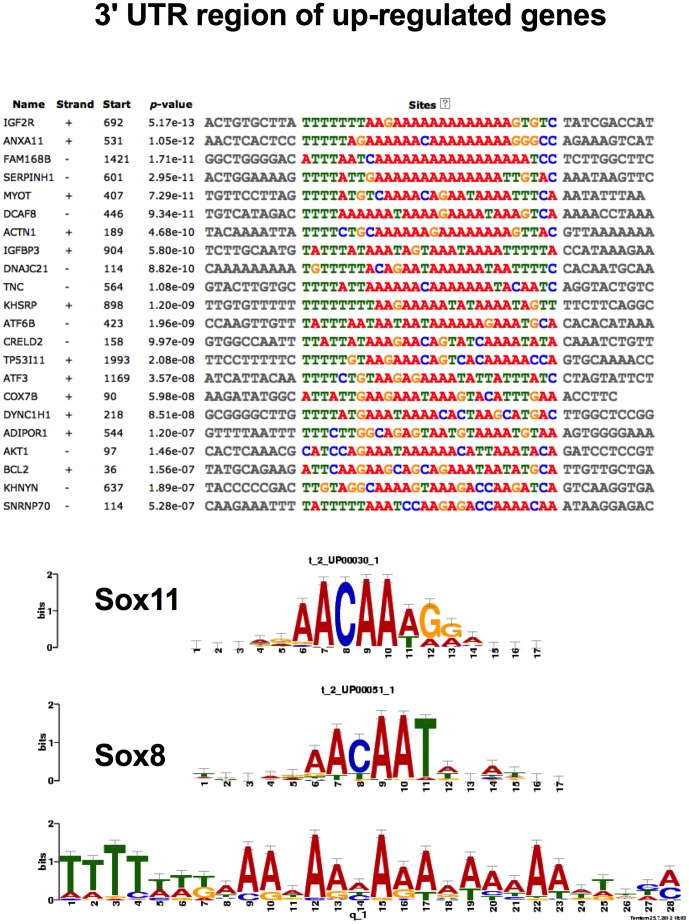
Demonstrates a common motif in the 3′ untranslated region of the upregulated genes. The bottom of the figure shows a cis-regulatory element for Sox family transcription factors, which can bind on the common motif of the genes shown above.

As shown in Figure 6, the 3′UTR of the upregulated genes demonstrated binding sites for the Sox family of genes. To our surprise, we were unable to identify any transcription factor binding site in the 3′ UTR common to the downregulated genes (data not shown).

### Analysis of the miRNA binding sites in the 3′UTR of the altered genes

Our analysis also demonstrated binding sites for several miRNA complementary to groups of upregulated and downregulated genes (Tables S1 & S2).

## Discussion

The present report is a continuation of our studies to understand fundamental mechanisms of LTH acclimatization responses in fetal sheep. In the present study, we identified a number of key genes, *cis*-regulatory elements, and miRNA that may play an important role in successful acclimatization of the ovine fetal cranial vasculature (and maybe other tissues) in association with exposure to *in-utero* long-term hypoxia (110 days). As noted, humans, rodents, and many other species show adverse effects in response to moderate prolonged high-altitude hypoxic exposure, whereas sheep appear to be more resilient. The mechanisms by which sheep and some other species successfully acclimatize to high-altitude LTH is not well-understood. Of importance, this may have implications in understanding these (responses, mechanisms) in humans to prevent of modify diseases such as acute/chronic mountain sickness, high altitude associated cerebral edema, or other conditions. Although a number of papers have reported on gene regulation in response to “chronic” hypoxia, almost without exception these exposures have been for a duration of only 48 hours or less [25]–[30]. Also, many of these studies are have been in cancer cell lines and demonstrate an increase in hypoxia inducible factor (HIF) 1α. Of critical importance, it is well documented that with chronic hypoxia HIF1α levels return to normoxic values within several days [31]. Similar to this study, we did not observed significant up-regulation of HIF1α with long-term hypoxia in the present study. Along this line, we have demonstrated that erythropoietin levels increased from 22.8±2.2 to 144±37 mu.ml^−1^ within 24 h; however, by day 7 it had returned to levels slightly above normal [32].

Also, our group has reported on alterations in fetal gene expression in response to LTH. In perirenal adipose tissue, these include: uncoupling protein 1, 11β hydroxy steroid dehydrogenase type 1, peroxisome proliferator activated receptor gamma coactivator 1, and deiodenase type 1 and 2 receptors [33]. In fetal adrenal zona fasciculata/reticularis these also include: nitric oxide synthase [34] phenylethanolamine-N methyltranferase, tyrosine hydroxylase, and dopamine β-hydroxylase [35]. Thus, LTH exposure during fetal life has a profound effect in modulating the expression of enzymes that mediate adrenomedullary catecholamine synthesis. In addition, these LTH-mediated responses include epigenetic-mediated repression of the estrogen receptor α gene in the uterine arteries [36]. Importantly, many of these genes are unaltered on hypoxia exposure in fetal carotid arteries, demonstrating tissue specific changes in gene expression.

This is first study to our knowledge to examine the effect of high-altitude (3801 m) long-term (110 days) hypoxia *in-utero* on global gene expression in the fetal vascular system. Importantly, we conducted this study in sheep that successfully acclimatize to LTH, escaping many of the problems associated with LTH exposure in humans and other species. For instance, in response to long-term (110 days) *in-utero* hypoxic exposure, sheep does not demonstrate intrauterine growth restriction, reduction in brain weight, alteration in CBF, and/or pulmonary hypertension. Therefore, the present study is of fundamental importance in identifying signaling molecules and pathways that play a protective role in acclimatization to high altitude LTH in the fetus of this species.

In the present study, we observed an increase in the gene expression for Insulin-like growth factor 2 receptor (IGF2R) and IGFBP3. A study in sheep with placental blood flow restriction by carunclectomy with superimposed hypoxia demonstrated IUGR with a failure to increase IGF2R [37]. In contrast, in the present study, we observed an increase in IGF2R without development of IUGR. We speculate that in the sheep fetus, IGF2R may be a vital factor providing protection hypoxia induced dysregulation in cerebral blood flow and brain growth restriction. Similarly, IGFBP3 may also be an important factor in prevention of dysregulated blood flow in the LTH exposed fetal sheep. Also, human studies demonstrate a significant lower IGFBP3 in growth restricted infants [38] as well as a significant increase in IGFBP3 with increased growth on treatment with growth hormone [39]. Thus, IGF2R and IGFBP3 both appear to be associated with protection from growth restriction in the sheep fetuses. Further investigations of these mechanisms are required, however.

We also observed upregulation of three members of the heat shock protein (HSP) 40 family. These were serpin peptidase inhibitor, DnaJ homolog subfamily C member 21, and DnaJ homolog subfamily B member 5 (Table 1). DnaJ/HSP40 proteins are conserved among phyla. They appear to play an important role in protein translation, folding, unfolding, translocation, and degradation, primarily by stimulating the ATPase activity of HSP70 chaperone proteins [40]. HSP47 is a 47 kDa collagen-binding glycoprotein localized in the endoplasmic reticulum. Gene ablation studies have indicated that HSP47 is essential for embryonic development and the maturation of several types of collagen [41]. However, further studies are needed to elucidate the role of these heat shock proteins in hypoxic acclimatization.

Also, in the present study, activating transcription factors (ATF) 3 and 6 were upregulated (Table 1). The mammalian ATF family of transcription factors represents a large group of basic region-leucine zipper (bZip) proteins; these can bind to cAMP-inducible transcription sites at a consensus sequence TGACGT(C/A)(G/A) [42]. These ATFs are important in stress signaling [43]. The present study suggests that these two factors may be important in fetal growth under hypoxic stress. Again, further investigation along this line is needed.

We also observed significant upregulation of solute carrier family 16 member 3 (Table 1), commonly known as monocarboxylic acid transporter 4 (MCT4). MCT4 (SLC16A3) is involved in the transport of metabolically important monocarboxylates such as lactate, pyruvate, acetate, and ketone bodies, and is important for lactate excretion to the extracellular space. It also assumes an important role during hypoxic stress [44]. Previously, upregulation of MCT4 by hypoxia has been demonstrated in the human bladder cancer transcriptome [45].

Other important genes upregulated with long-term hypoxia include AKT1, BCL2, and TGFβ3 (Table 1; Figure 1). AKT1 has been shown to be crucial in cell survival on hypoxic exposure, and failure to up-regulate AKT1 is involved in hypoxia-induced cell death [46]. Of importance, AKT1 knockout mice are growth restricted [47]. Thus, AKT1 may be another important protective molecule playing a role in normal birth weight of newborn lambs, despite their long-term *in-utero* hypoxic exposure. Additionally, B-cell lymphoma 2 (BCL2) proteins are critical regulators of cell death, whose main function is to regulate the release of cytochrome c from mitochondria in the intrinsic apoptotic pathway [48]. Importantly, BCL2 is known to be reduced in intrauterine growth restriction [49], and upregulation of BCL2 in our studies may play a protective role against LTH for fetal lambs. Moreover, as shown by other studies, BCL2 upregulation is important in cell survival during prolonged hypoxic exposure [50]. Similarly, TGFβ3 has been shown to be upregulated with hypoxia [51]. The role of these proteins in preventing adverse long-term hypoxia effects needs to be addressed.

In contrast to upregulation, we found a limited number of genes to be downregulated because of antenatal LTH exposure (Table 2). These included brain ribonucleas (BRN-2), which has been shown to be downregulated in perinatal asphyxia in rat brain [52]. We also observed significant reduction in arachndonate 5-lipoxygenase-activating protein (ALOX5AP). ALOX5AP is a non-heme iron containing dioxygenase which incorporates oxygen into arachidonic acid and mediates leukotriene formation [53]. This gene has been shown to be induced by hypoxia and is important in chronic hypoxia-induced vascular remodeling and pulmonary hypertension [53], [54]. Instead of upregulation, as observed in the quoted studies, we observed LTH-induced downregulation of ALOX5AP. In addition, this contrast, with our antenatal maternal LTH exposed newborn lambs, in which pulmonary hypertension did not develop [55]. It appears that sheep can alter multiple molecules, which protect the fetus from reduced brain growth dysregulation of CBF, as well as pulmonary hypertension.

Another important gene downregulated with LTH was cellular retinoic acid binding protein 2 (CRABP2; Table 2). This chaperone protein can bind intracellular retinoic acid with high affinity, and translocate it to the nucleus to induce cellular differentiation [56]. CRABP2 downregulation, may relieve the negative inhibition of retinoic acid signaling on angiogenesis [57]. We also observed a significant downregulation of ribosomal protein L35 (RPL35), a conserved protein in eukaryotes important in ribosome biogenesis [58]. Reduced levels of RPL35 are associated with delay of the G1 phase of cell cycle [58]. Its exact role in long-term hypoxia is not known, however.

Importantly, the question arises, by what mechanisms does LTH alter so many genes in a highly orchestrated manner? Through common *cis*-regulatory elements and *trans*-acting factors, prolonged hypoxia appears to lead to the upregulation of several of these genes belonging to different canonical and functional pathways. To address this issue, we examined promoters and 3′ UTRs of the altered genes to find common *cis*-regulatory elements and sites complementary to miRNAs. We have identified the Pbx/Knotted homeobox binding site in the promoter region of a number of upregulated genes. This agrees with the finding in skeletal muscle, in which chronic hypoxia leads to conversion of fast to slow fibers with increased expression of Pbx/Knotted homeobox [59]. Pbx also has been shown to be involved in cardiac muscle differentiation [60]. Moreover, Pbx1 silencing impairs endothelial cell migration and blocks angiogenesis [61]. Also, in the 3′ UTR of the number of upregulated genes, we observed several binding sites for the Sox family transcription factors important in the development of fetal vasculogenesis [62] and postnatal angiogenesis [63]. Further experiments are needed, however, to establish the role of Pbx1 in the regulation of angiogenesis pathways such as VEGF and PI3/AKT, and their role in LTH acclimatization. The present report provides strong rationale for such studies.

Furthermore, in the promoter region of the downregulated genes we observed binding sites for DOT6 and TOD6, components of the RPD3 histone deacetylase complex RPD3C(L) responsible for the deacetylation of lysine residues on the N-terminal part of the chromatin core histones (H2A, H2B, H3 and H4) [64]. Of importance, histone deacetylation may lead to epigenetic repression and play an important role in transcriptional regulation, cell cycle progression, and developmental events. DOT6 and TOD6 bind to sequences containing the core CGATG, which resembles the PAC (Polymerase A and C) motif [64]. Thus, these studies indicate that chronic hypoxia may lead to DOT6-mediated histone deacetylation to achieve gene downregulation. These findings also require further investigation, however.

We also analyzed the up- and downregulated genes for putative miRNA binding sites (Table S1 & Table S2). We have identified miRNA with putative binding sites to a number of altered genes. Again, confirmation of these miRNA will require future studies.

## Speculation and Perspective

On exposure to antenatal long-term hypoxia, humans and other species demonstrate fetal intrauterine growth restriction, dysregulation of CBF, pulmonary hypertension, and other changes. Sheep does not, however. Also, previously, we have shown that in response to *in-utero* LTH exposure, fetal lambs develop neither dysregulation of cerebral blood flow nor pulmonary hypertension. The present study provides insight into these mechanisms. However, the genes altered may simply be result of long-term hypoxic exposure and may be detrimental instead of adaptive. The limitation of the present study is that it does not causally examine the effect of the microarray data with functional implications. However, such studies are beyond the scope of the present study and need to be confirmed independently. The present study provides a number of molecules associated with acclimatization to high altitude as possible for such studies

## Supporting Information

Table S1 **Putative miRNA complementary to the 3′ UTR of the upregulated genes in high altitude acclimatized fetal carotid arteries compared to normal control.**
(DOC)

Table S2 **Putative miRNA complementary to the 3′ UTR of the downregulated genes in high altitude acclimatized fetal carotid arteries compared to normal control.**
(DOC)
