# Supplementary material for: Antenatal Maternal Long-Term Hypoxia: Acclimatization Responses with Altered Gene Expression in Ovine Fetal Carotid Arteries
Source: PLoS One. 2013 Dec 18;8(12):e82200. doi: 10.1371/journal.pone.0082200 (PMC3867347; doi:10.1371/journal.pone.0082200)
Supplement: Table S1 — Putative miRNA complementary to the 3′ UTR of the upregulated genes in high altitude acclimatized fetal carotid arteries compared to normal control. (DOC) [file pone.0082200.s001.doc]

Table S1- Putative miRNA complementary to the 3' UTR of the upregulated genes in high altitude acclimatized fetal carotid arteries compared to normal control

| miRNA | Gene |
| --- | --- |
| hsa-miR-657; hsa-mir-940 | ACTN1 |
|  | AKT1 |
|  | ANXA11 |
|  | ATF3 |
|  | BCL2 |
|  | CDV3 |
|  | COL18A1 |
|  | DNAJB5 |
|  | FADS2 |
|  | HR |
|  | IGF2R |
|  | IGFBP3 |
|  | KHSRP |
|  | MED16 |
|  | MTCH1 |
|  | MTMR2 |
|  | PIM2 |
|  | PLXNB1 |
|  | RBM42 |
|  | SERPINH1 |
|  | TP53I11 |
| hsa-miR-449b; hsa-miR-940; hsa-miR-34c-5p; hsa-mir-885-3p | ACTN1 |
|  | ANXA11 |
|  | BCL2 |
|  | CDV3 |
|  | COL18A1 |
|  | CRELD2 |
|  | DNAJB5 |
|  | FADS2 |
|  | HR |
|  | IGFBP3 |
|  | KHSRP |
|  | MED16 |
|  | MTCH1 |
|  | MTMR2 |
|  | PIM2 |
|  | PLXNB1 |
|  | SERPINH1 |
|  | SURF6 |
|  | TGFB3 |
|  | TP53I11 |
