# Supplementary material for: Antenatal Maternal Long-Term Hypoxia: Acclimatization Responses with Altered Gene Expression in Ovine Fetal Carotid Arteries
Source: PLoS One. 2013 Dec 18;8(12):e82200. doi: 10.1371/journal.pone.0082200 (PMC3867347; doi:10.1371/journal.pone.0082200)
Supplement: Table S2 — Putative miRNA complementary to the 3′ UTR of the downregulated genes in high altitude acclimatized fetal carotid arteries compared to normal control. (DOC) [file pone.0082200.s002.doc]

Table S2-Putative miRNA complementary to the 3' UTR of the downregulated genes in high altitude acclimatized fetal carotid arteries compared to normal control

| miRNA | Gene |
| --- | --- |
| hsa-mir-298 | ALOX5AP |
|  | BATF3 |
|  | RNASE6 |
| hsa-mir-330-3p | FAM35A |
|  | RNASE6 |
|  | RGS10 |
|  | CRABP2 |
| hsa-mir-922 | BATF3 |
|  | FAM35A |
|  | RNASE6 |
